# Exploring the mediating effects of amino acids on BMI and gestational diabetes mellitus: a longitudinal population-based cohort study

**DOI:** 10.3389/fendo.2025.1502678

**Published:** 2025-05-20

**Authors:** Lijuan Wang, Haiyan Wang, Xiaoyan Gou, Pengsheng Li, Dazhi Fan, Fang Huang, Dandan Yu, Xingni Yang, Dongmei Suo, Zhengping Liu, Gengdong Chen, Zixing Zhou

**Affiliations:** ^1^ Department of Obstetrics, Foshan Women and Children Hospital, Foshan, China; ^2^ Foshan Fetal Medicine Research Institute, Foshan Women and Children Hospital, Foshan, China

**Keywords:** amino acid metabolism, glucose, BMI, gestational diabetes mellitus, mediating effect, cohort study

## Abstract

**Objective:**

To investigate the associations between pre-pregnancy BMI, amino acid metabolism in early pregnancy, and glucose levels/gestational diabetes mellitus (GDM) in later pregnancy. We also examined the mediating effects of amino acids on BMI and glucose.

**Methods:**

The cohort study examined the association between BMI, first-trimester amino acids, and glucose/GDM among 1074 pregnant women. Regression analyses detected changes in amino acid levels and glucose measurements from oral glucose tolerance test (OGTT) at the 10th, 50th, and 90th percentiles. A mediation analysis was conducted to determine if amino acids mediated the relationship between BMI and glucose/GDM.

**Results:**

Four essential amino acid concentrations (leucine, phenylalanine, threonine, and valine) increased significantly with increasing BMI (P < 0.05). Additionally, overweight women exhibited higher levels of non-essential amino acids (alanine, arginine, asparagine, aspartate, proline, tyrosine) and ornithine than underweight and normal-weight women. Women with GDM demonstrated higher levels of leucine, valine, alanine, asparagine, proline, and tyrosine compared to those without (P < 0.05). Fasting blood glucose (OGTT0) increased by 0.07 mmol/L when alanine levels increased by 50%. Similarly, increasing asparagine and leucine levels by 50% led to a 0.24 mmol/L increase in 1-hour postprandial blood glucose (OGTT1). A 50% rise in alanine, asparagine, and leucine levels led to average increases of 0.31, 0.19, and 0.21 mmol/L in mean 2-h postprandial blood glucose (OGTT2). These associations were statistically significant at the upper 90th percentile of the OGTT2 distribution. 50% increase in valine was correlated with a 0.22 mmol/L increase in mean OGTT2. The levels of alanine accounted for 11.76%, 8.08%, and 11.38% of the associations between BMI and GDM, OGTT0, and OGTT2, respectively. Additionally, the indirect effect of BMI-associated OGTT2 on leucine levels was estimated to be 5.39 percent.

**Conclusion:**

Amino acid metabolism is correlated with BMI, GDM, and glucose levels. Notably, BMI and GDM/glucose intolerance are significantly mediated by alanine and leucine levels. This suggests a new way to study why overweight or obese mothers are more likely to develop GDM and glucose intolerance.

## 1 Introduction

Gestational diabetes mellitus (GDM) is a prevalent obstetric complication, affecting approximately 14.8% of pregnant women in China (1), and giving rise to a range of detrimental consequences (2). The etiology and underlying pathophysiological mechanisms of GDM cannot be fully elucidated solely by established risk factors, such as advanced maternal age (3). Consequently, it is imperative to conduct a comprehensive investigation into the pathogenesis of GDM, considering multiple factors.

The link between pre-pregnancy overweight/obesity and gestational diabetes, along with other metabolic diseases, is well-known. These conditions also negatively impact pregnancy and maternal health (4). To reduce these risks, guidelines recommend enhanced antenatal care for obese women (5, 6). Insulin resistance(IR), marked by a reduced insulin response and impaired glucose regulation, is crucial in developing GDM and is closely linked to obesity (7). A recent study using birth certificate data from 2013 to 2018 shows increasing risks of adverse outcomes due to obesity (8). Therefore, managing weight before pregnancy is vital for preventing GDM. Additionally, exploring GDM’s causes by combining weight with new factors is essential.

Metabolomics allows for the simultaneous analysis of a vast array of metabolites, facilitating a high-throughput evaluation of the metabolic state of a biospecimen. Research has shown that the study of amino acid metabolism can unveil novel biomarkers for metabolic disorders, such as GDM (9, 10). Specifically, branched-chain amino acids (BCAAs) and aromatic amino acids (AAAs) have been implicated in the development, progression, and remission of insulin resistance, diabetes, and obesity (11, 12).The levels of alanine, isoleucine, and tyrosine in early pregnancy exhibited significant variations between Chinese women who developed GDM and those who did not, as reported in a study (9). However, another case-control study failed to identify any differences in concentrations of BCAAs during early pregnancy between women with and without GDM (13, 14). Furthermore, recent research has shed light on the associations between body weight, amino acid metabolism, and diabetes. It has been found that higher BCAA intake is linked to increased risks of overweight and insulin resistance in children born to mothers with GDM (15). The study revealed that obese women with GDM had higher levels of BCAAs (valine, leucine, and isoleucine) during early pregnancy compared to non-obese women with GDM (16). However, the association between the metabolic profile of amino acids, particularly in the early stages of pregnancy, and the development of GDM remains unclear. Additionally, the interplay between amino acids, body weight, and glucose levels has yet to be fully elucidated. Therefore, a prospective study investigating the pre-pregnancy weight, amino acid profile during early pregnancy, and the incidence of GDM in pregnant women is warranted.

Based on the observed correlation between weight, amino acids, and GDM, our hypothesis posits that the metabolic profile of amino acids during the first trimester of early pregnancy is associated with pre-pregnancy BMI and GDM. Furthermore, we propose that amino acids during the first trimester mediate the relationship between pre-pregnancy BMI and GDM. This hypothesis is based on the premise that pre-pregnancy overweight or obesity leads to persistent metabolic disturbances, which in turn influence early-pregnancy amino acid profiles that contribute to the pathogenesis of GDM. Although amino acid levels were assessed in early pregnancy, pre-pregnancy BMI serves as an indicator of chronic metabolic conditions that may continuously affect gestational metabolism through hormonal pathways or adipose-derived factors. To investigate this relationship further, we conducted a prospective study with an expanded sample size.

## 2 Subjects

The birth cohort study, a cohort study conducted in Foshan, China, spanned from March 2020 to April 2021. Pregnant women were enlisted during their initial obstetric clinic visit at the Foshan Women and Children’s Hospital. Approval for the study was obtained from the Human Subjects Committee of the hospital. The inclusion criteria encompassed singleton pregnancy, absence of pre-pregnancy diabetes, and gestational age in the first trimester (<14 weeks).The exclusion criteria encompassed three factors: (1) the absence of data pertaining to the detection of amino acids metabolism, (2) the absence of data regarding the oral glucose tolerance test (OGTT) conducted between 24 and 28 weeks of gestation, and (3) the presence of acute and chronic diseases, including hypertension, heart disease, liver disease, and kidney disease (excluding polycystic ovary syndrome (PCOS)). Ultimately, a total of 1074 women were included in the study.

China exhibits a high prevalence of PCOS among women of reproductive age, with a significant proportion achieving successful pregnancies following treatment. Consequently, the inclusion of pregnant women with PCOS in this study does not compromise the generalizability of the findings; rather, it enhances it. To mitigate heterogeneity, PCOS has been incorporated as a covariate in our statistical analyses.

## 3 Material and methods

### 3.1 Data collection and variables’ definition

To gather data on maternal demographic characteristics, a face-to-face survey was administered to pregnant women. The OGTT data was obtained from computerized databases within the hospital. Maternal demographic information encompassed variables such as maternal age, parity, level of education, family income, family history of diabetes, polycystic ovary syndrome, current smoking, passive smoking, current drinking, folic acid supplements prepare for pregnancy, pre-pregnancy BMI, fasting blood for amino acid metabolism in the first trimester (<14 weeks), weight gain during pregnancy before OGTT, and gestational age for amino acid metabolism detection. To ensure patient confidentiality, all identifiable patient information was de-identified. Specially, The pre-pregnancy BMI data for our participants were obtained retrospectively, based on their recollection during the first trimester birth examination.

GDM was diagnosed utilizing the criteria established by the International Association for Diabetes in Pregnancy Study Group (IADPSG). Pregnant women underwent OGTT between 24 and 28 weeks of gestation. The results were classified based on specific cut-off points: fasting plasma glucose (FPG, OGTT0) ≥5.1 mmol/L, 1-hour postprandial plasma glucose (1-h PG, OGTT1) ≥10.0 mmol/L, or 2-hour postprandial plasma glucose (2-h PG, OGTT2) ≥8.5 mmol/L (17). Pre-pregnancy BMI in kg/m^2^ was categorized into three groups according to the Chinese BMI criteria for the general adult population: BMI < 18.5 kg/m^2^, 18.5 kg/m^2^≤ BMI < 24 kg/m^2^, and BMI ≥ 24 kg/m^2^ (18).

### 3.2 Measurement of blood amino acids

Blood amino acid metabolism consists of a total of eight essential amino acids, namely histidine (His), leucine (Leu), lysine (Lys), methionine (Met), phenylalanine (Phee), threonine (Thr), tryptophan (Trp), and valine (Val). Additionally, there are eleven non-essential amino acids, including alanine (Ala), arginine (Arg), asparagine (Asn), aspartate (Asp), cysteine (Cys), glutamine (Gln), glutamate (Glu), glycine (Gly), proline (Pro), serine (Ser), and tyrosine (Tyr). Furthermore, there are four amino acid metabolites, namely citrulline (Cit), homocysteine (Hcy), ornithine (Orn), and pipercide (Pip). Peripheral venous blood samples were collected within the obstetric clinic and subsequently transferred to the Department of Laboratory Medicine at the hospital for the purpose of amino acid metabolism detection. This analysis was conducted using the liquid chromatography-tandem mass spectrometry method (10, 19) (LC-MS/MS API 3200 MDTM, AB SciexPte.Ltd; LC-20AD, Shimadzu). Due to the limitations of the testing equipment available in our hospital, which did not include isoleucine in its test panel, we were unable to assess this amino acid. We plan to incorporate isoleucine detection in future related studies.

### 3.3 Statistical analysis

The mean ± SD or number (%) was reported for the baseline characteristics. In order to examine amino acid metabolism, the ANOVA method was employed to compare BMI groups, while the T-test was utilized to compare women with and without GDM. Additionally, multiplicative interaction effects between BMI and amino acids on GDM were identified. For the analysis of GDM, amino acid levels were stratified into tertiles. Binary logistic regression was conducted to examine the association between amino acids and GDM in both crude and adjusted models. In adjusted model, the confounding variables were maternal age, parity, level of education, family income, family history of diabetes, polycystic ovary syndrome, passive smoking, folic acid supplements prepare for pregnancy, pre-pregnancy BMI, fasting blood for amino acid metabolism, weight gain during pregnancy before OGTT, and gestational age for amino acids metabolism detection. The statistical software SPSS 24.0 was employed to conduct the analyses.

Quantile regression analysis was utilized to investigate potential changes in the distribution of three-hour glucose levels during the oral glucose tolerance test (OGTT) and to assess the relationship between amino acid levels and extreme values of glucose. Quantile regression analysis was employed to elucidate how amino acids differentially influence glucose levels across its distribution. Analyzing 10th, 50th, and 90th percentiles is not a multiple testing issue but an exploration of effect heterogeneity. Quantile regression does not require multiple correction and is a whole regression analysis in itself. We chose restricted cubic spline (RCS) knots for their ability to assess nonlinear associations between metabolites and GDM risk using logistic regression. The knots were placed at the 10th, 50th, and 90th percentiles to balance flexibility and prevent overfitting, following epidemiological guidelines (20). The 10th percentile served as the reference for odds ratio (OR) calculations. Linear regression models were established for each percentile and the mean of glucose. Based on the 1.5 logarithm function, a log transformation was applied to each amino acid levels to ensure that a 1-unit change on the transformed scale fell within or near the interquartile range (IQR). It should be noted that a 1-unit difference on the natural log-transformed scale encompasses a much wider range than the IQR on the original scale (21). The models were adjusted for various factors including maternal age, parity, level of education, family income, family history of diabetes, polycystic ovary syndrome, passive smoking, folic acid supplements prepare for pregnancy, pre-pregnancy BMI, fasting blood for amino acid metabolism, weight gain during pregnancy before OGTT, and gestational age for amino acids metabolism detection. We adjusted confounders using the “Enter” method. Quantile regression was conducted in the analyses using R version 3.5.2, along with the quantreg and forestplot packages.

We explored the mediating effect of amino acid on pre-pregnancy BMI and glucose or GDM in further. Pre-pregnancy BMI indicates chronic metabolic conditions, while first-trimester amino acids show early pregnancy adaptations that might lead to later glucose issues. The robust maximum-likelihood estimation was used for mediation analysis, one of the models of structural equation modeling. To estimate the standardized coefficients of the direct, indirect, and total effects, we utilized the bootstrap method with 2,000 resamples. Additionally, we adjusted our models for various confounding factors, including maternal age, parity, level of education, family income, family history of diabetes, polycystic ovary syndrome, passive smoking, folic acid supplements prepare for pregnancy, fasting blood for amino acid metabolism, weight gain during pregnancy before OGTT, and gestational age for amino acids metabolism detection. We adjusted confounders using the “Enter” method. We calculated the mediation proportion of indirect effect as follows: Estimated mediated = Indirect effect/Total effect×100%. Total effect = Direct effect + Indirect effect. The data analysis was conducted using Mplus 7.4 (Muthén and Muthén). Statistical significance was defined as a two-sided P value less than 0.05.

## 4 Results

### 4.1 Baseline characteristics

**Table 1** summarizes characteristics of the study population, including average age of mothers (29.27 years), percentage of primipara (42.83%), family history of diabetes (8.10%), polycystic ovary syndrome (5.40%), passive smoking exposure (25.79%), folic acid supplement intake (30.82%), BMI in first trimester (20.63 kg/m2), and weight gain before OGTT (7.11 kg).

**Table 1:** The baseline characteristics of study population in the cohort study.

Characteristic	Total (n = 1074)
Maternal age (mean ± SD, years)	29.27 ± 4.41
Primipara (n, %)	460 (42.83)
Level of education (n, %)	
High school and below	447 (41.62)
Junior college and below	627 (58.38)
Income (n, %)	
< 5000 yuan	369 (36.87)
5000 ≤ BMI < 10000 yuan	481 (44.79)
≥ 10000 yuan	224 (20.86)
Family history of diabetes (n, %)	87 (8.10)
Polycystic ovary syndrome (n, %)	58 (5.40)
Current smoking (n, %)	0
Passive smoking (n, %)	277 (25.79)
Current drinking (n, %)	0
Folic acid supplements prepare for pregnancy (n, %)	331 (30.82)
Pre-pregnancy BMI(mean ± SD, Kg/m^2^)	20.63 ± 2.84
BMI < 18.5 (n, %)	256 (23.84)
18.5 ≤ BMI < 24 (n, %)	693 (64.52)
BMI ≥ 24 (n, %)	125 (11.64)
Gestational age for amino acid metabolism (mean ± SD, weeks)	10.19 ± 1.63
Fasting blood for amino acid metabolism(n, %)	
Yes	126 (11.73)
No	743 (69.18)
Unknown	205 (19.09)
GDM (n, %)	202 (18.81)
OGTT (mean ± SD, mmol/L)	
OGTT0	4.41 ± 0.42
OGTT1	7.87 ± 1.85
OGTT2	6.87 ± 1.52
Weight gain during pregnancy before OGTT (mean ± SD, kg)	7.11 ± 3.72

GDM, gestational diabetes mellitus; SD, standard deviation; BMI, body mass index; OGTT, oral glucose tolerance test. OGTT0, fasting plasma glucose; OGTT1, 1-h postprandial plasma glucose; OGTT2, 2-h postprandial plasma glucose.

### 4.2 Differences of glucose and amino acids based on BMI

A strong association was found between BMI and fasting blood glucose, postprandial blood glucose, and incidence of GDM (P<0.001) (**Table 2**). Higher BMI was linked to increased levels of essential amino acids Leu, Phe, Thr, and Val (P<0.05). Furthermore, the three BMI groups exhibited significant differences in six non-essential amino acids (P<0.05). Specifically, as BMI increased, the levels of Ala, Asn, Pro, and Tyr also increased. Women with a BMI ≥ 24 had significantly higher concentrations of Arg and Asp compared to those with a BMI < 18.5 and 18.5 ≤ BMI < 24. Orn levels also increased with higher BMI (P=0.026).

**Table 2:** Differences of glucose and amino acids based on BMI.

Distribution (n, %) or (mean ± SD)	BMI<18.5 (n = 256)	18.5 ≤ BMI<24 (n = 693)	BMI ≥ 24 (n = 125)	P-value
GDM	31 (12.11)	129 (18.61)	42 (33.60)	< 0.001**
OGTT0 (mmol/L)	4.36 ± 0.33	4.44 ± 0.37	4.60 ± 0.68	< 0.001**
OGTT1 (mmol/L)	7.34 ± 1.68	7.80 ± 1.77	8.81 ± 2.25	< 0.001**
OGTT2 (mmol/L)	6.40 ± 1.29	6.93 ± 1.44	7.50 ± 2.02	< 0.001**
Essential amino acid (μmol/L)				
His	123.40 ± 58.70	129.50 ± 75.99	125.45 ± 49.75	0.456
Leu	91.97 ± 23.88	94.59 ± 22.93	101.14 ± 25.61	0.002*
Lys	24.87 ± 22.42	25.70 ± 25.29	27.51 ± 24.69	0.617
Met	16.37 ± 4.61	16.28 ± 4.28	16.32 ± 3.61	0.963
Phe	43.93 ± 8.84	45.80 ± 8.76	47.88 ± 11.78	< 0.001**
Thr	14.60 ± 3.38	15.53 ± 3.41	16.35 ± 3.48	< 0.001**
Trp	52.43 ± 11.45	51.94 ± 12.83	50.72 ± 9.83	0.437
Val	146.57 ± 27.36	152.35 ± 27.38	159.85 ± 27.39	< 0.001**
Non-essential amino acid (μmol/L)				
Ala	139.24 ± 25.49	147.72 ± 27.77	156.01 ± 27.68	< 0.001**
Arg	16.09 ± 8.55	15.41 ± 7.03	17.20 ± 7.31	0.035*
Asn	14.67 ± 3.49	15.30 ± 3.67	16.46 ± 3.95	< 0.001**
Asp	36.70 ± 11.63	36.61 ± 10.86	39.33 ± 14.30	0.048*
Cys	0.47 ± 0.17	0.47 ± 0.20	0.45 ± 0.14	0.516
Gln	27.57 ± 25.75	28.49 ± 29.09	30.47 ± 27.65	0.645
Glu	78.74 ± 14.53	79.45 ± 14.50	80.89 ± 14.55	0.397
Gly	85.22 ± 18.49	86.28 ± 19.21	85.37 ± 19.67	0.704
Pro	421.18 ± 121.19	449.62 ± 130.01	469.85 ± 137.05	0.001*
Ser	71.73 ± 16.39	71.78 ± 17.21	73.31 ± 21.16	0.650
Tyr	29.65 ± 8.15	30.14 ± 7.65	33.14 ± 8.19	< 0.001**
Metabolism of amino acid (μmol/L)				
Cit	14.66 ± 4.17	14.40 ± 3.37	14.47 ± 3.31	0.604
Hcy	13.93 ± 1.46	13.76 ± 1.45	13.66 ± 1.58	0.172
Orn	18.95 ± 4.26	19.43 ± 4.35	20.24 ± 4.79	0.026*
Pip	95.63 ± 19.78	95.47 ± 20.25	94.76 ± 18.62	0.919

ANOVA method was employed to compare BMI groups, *P<0.05, **P<0.001. GDM, gestational diabetes mellitus; SD, standard deviation; BMI, body mass index; OGTT, oral glucose tolerance test. OGTT0, fasting plasma glucose; OGTT1, 1-h postprandial plasma glucose; OGTT2, 2-h postprandial plasma glucose. The bold in the table means statistically significant (P < 0.05).

### 4.3 Association between amino acids and glucose

Regarding essential amino acids, women with GDM exhibited significantly elevated levels of Leu and Val compared to those without GDM (mean 99.06 vs. 93.72μmol/L, P=0.004; mean 157.46 vs. 150.54μmol/L, P=0.001, respectively). A notable distinction was observed between women with and without GDM concerning the non-essential amino acids Ala, Asn, Pro, and Tyr (mean 153.64 vs. 145.04μmol/L, P<0.001; mean 15.98 vs. 15.13μmol/L, P=0.003; mean 476.01 vs. 438.05μmol/L, P<0.001; mean 31.48 vs. 30.12μmol/L, P=0.028, respectively) (**Table 3**). Participants were categorized into three subgroups based on BMI, and we examined the variations in amino acid levels between pregnant women with GDM and those without, across different BMI subgroups. The analysis revealed that the disparities in six amino acids (leucine, valine, alanine, asparagine, proline, and tyrosine) between GDM and non-GDM pregnant women were predominantly observed in individuals with normal BMI (P-values were 0.004, 0.001, 0.005, 0.008, 0.002, and 0.032, respectively) (**Supplementary Table A1**). Furthermore, when these six amino acids were categorized into tertiles (**Supplementary Table A2**), the findings remained consistent, demonstrating that all six amino acids were significantly associated with GDM after statistical adjustment.

**Table 3:** Differences of amino acids between GDM and non-GDM groups.

Amino acids (mean ± SD)	Non-GDM	GDM	P-value
Essential amino acid (μmol/L)			
His	128.52 ± 72.06	123.51 ± 57.38	0.357
Leu	93.72 ± 23.56	99.06 ± 23.34	0.004*
Lys	25.51 ± 23.98	26.60 ± 26.96	0.570
Met	16.25 ± 4.20	16.54 ± 4.63	0.394
Thr	15.35 ± 3.38	15.63 ± 3.59	0.293
Trp	52.07 ± 12.54	51.27 ± 10.59	0.405
Val	150.54 ± 27.00	157.46 ± 29.50	0.001*
Non-essential amino acid (μmol/L)			
Ala	145.04 ± 27.25	153.64 ± 28.34	< 0.001**
Arg	15.87 ± 7.50	15.37 ± 7.34	0.386
Asn	15.13 ± 3.69	15.98 ± 3.61	0.003*
Asp	37.22 ± 11.57	35.74 ± 11.25	0.099
Cys	0.46 ± 0.19	0.48 ± 0.15	0.240
Gln	28.33 ± 27.42	29.57 ± 31.15	0.573
Glu	79.35 ± 14.32	79.90 ± 15.33	0.628
Gly	85.91 ± 18.52	85.98 ± 21.39	0.961
Phe	45.40 ± 9.25	46.45 ± 8.82	0.141
Pro	438.05 ± 125.64	476.01 ± 141.45	< 0.001**
Ser	72.16 ± 17.45	71.01 ± 17.82	0.403
Tyr	30.12 ± 7.98	31.48 ± 7.44	0.028*
Metabolism of amino acid (μmol/L)			
Cit	14.41 ± 3.63	14.73 ± 3.31	0.241
Hcy	13.81 ± 1.46	13.70 ± 1.50	0.365
Orn	19.50 ± 4.42	19.01 ± 4.25	0.154
Pip	95.50 ± 19.67	95.09 ± 21.08	0.791

T-test was utilized to compare women with and without GDM. *P<0.05, **P<0.001. GDM, gestational diabetes mellitus; SD, standard deviation; BMI, body mass index; OGTT, oral glucose tolerance test. The bold in the table means statistically significant (P < 0.05).

**Figure 1:**
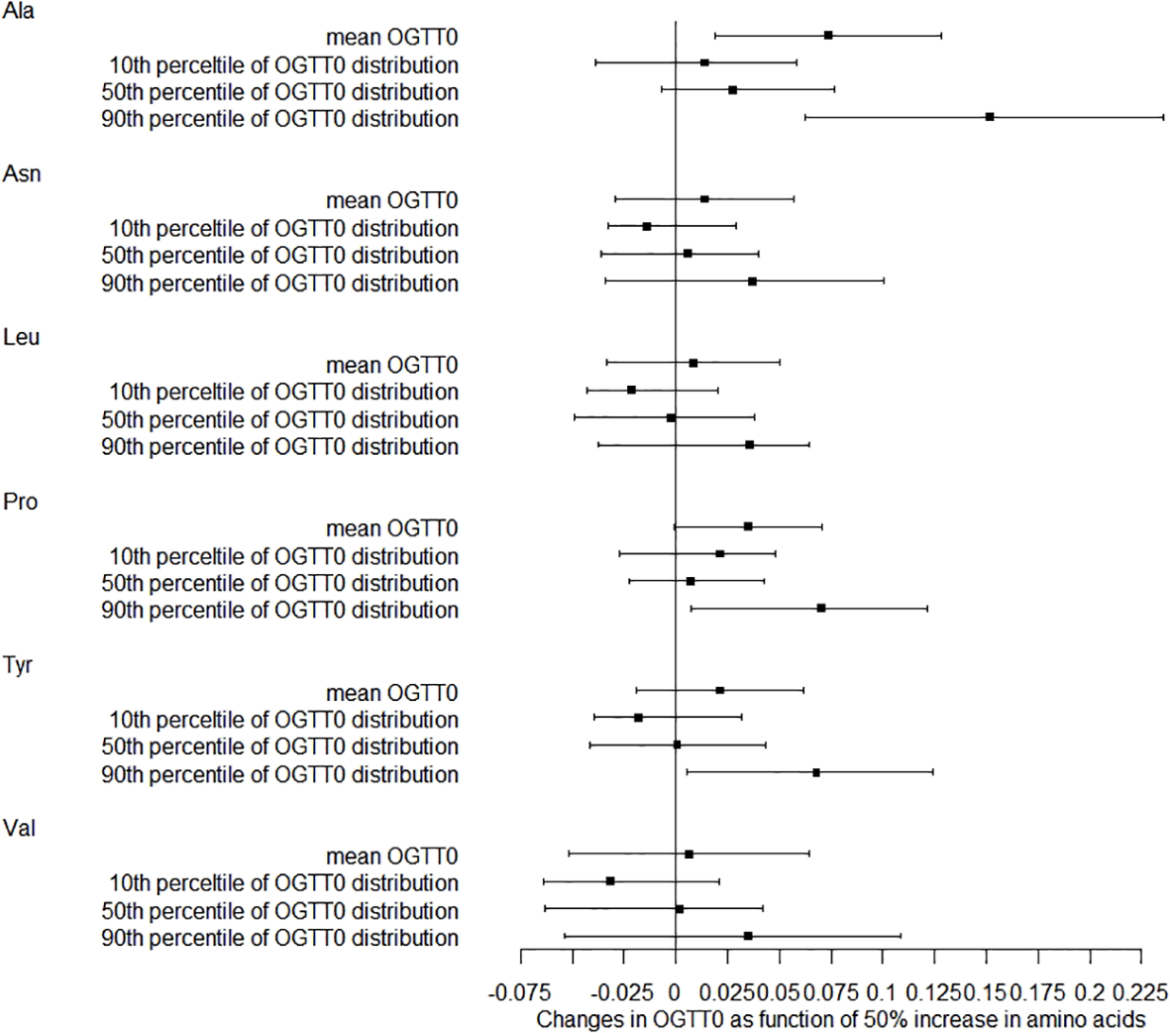
Distribution changes in OGTT0 as function of 50% increase in amino acids. The models were adjusted for maternal age, parity, level of education, family income, family history of diabetes, polycystic ovary syndrome, passive smoking, folic acid supplements prepare for pregnancy, pre-pregnancy BMI, fasting blood for amino acid metabolism, weight gain during pregnancy before OGTT, gestational age for amino acids metabolism detection.

**Figure 2:**
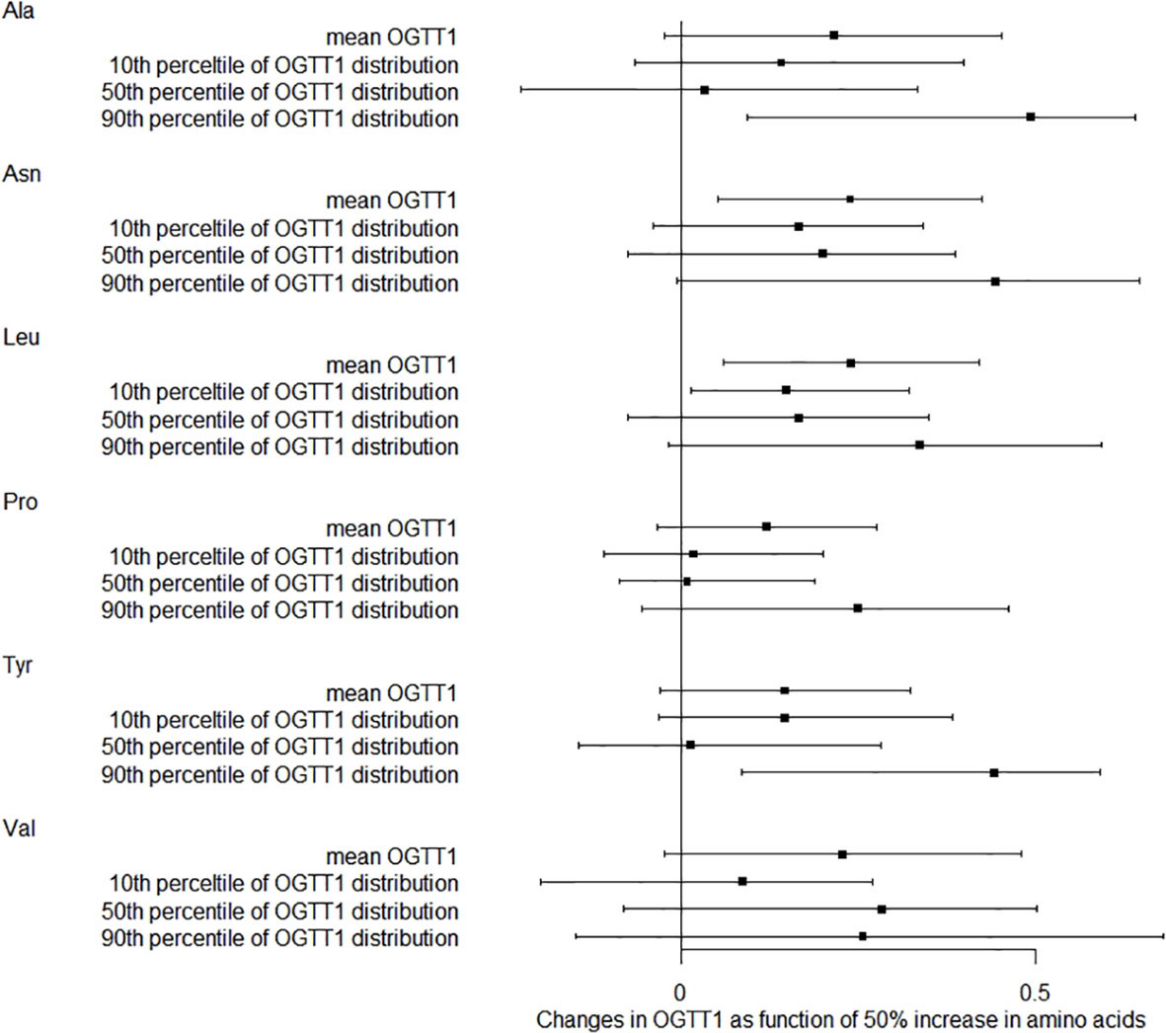
Distribution changes in OGTT1 as function of 50% increase in amino acids. The models were adjusted for maternal age, parity, level of education, family income, family history of diabetes, polycystic ovary syndrome, passive smoking, folic acid supplements prepare for pregnancy, pre-pregnancy BMI, fasting blood for amino acid metabolism, weight gain during pregnancy before OGTT, gestational age for amino acids metabolism detection.

**Figure 3:**
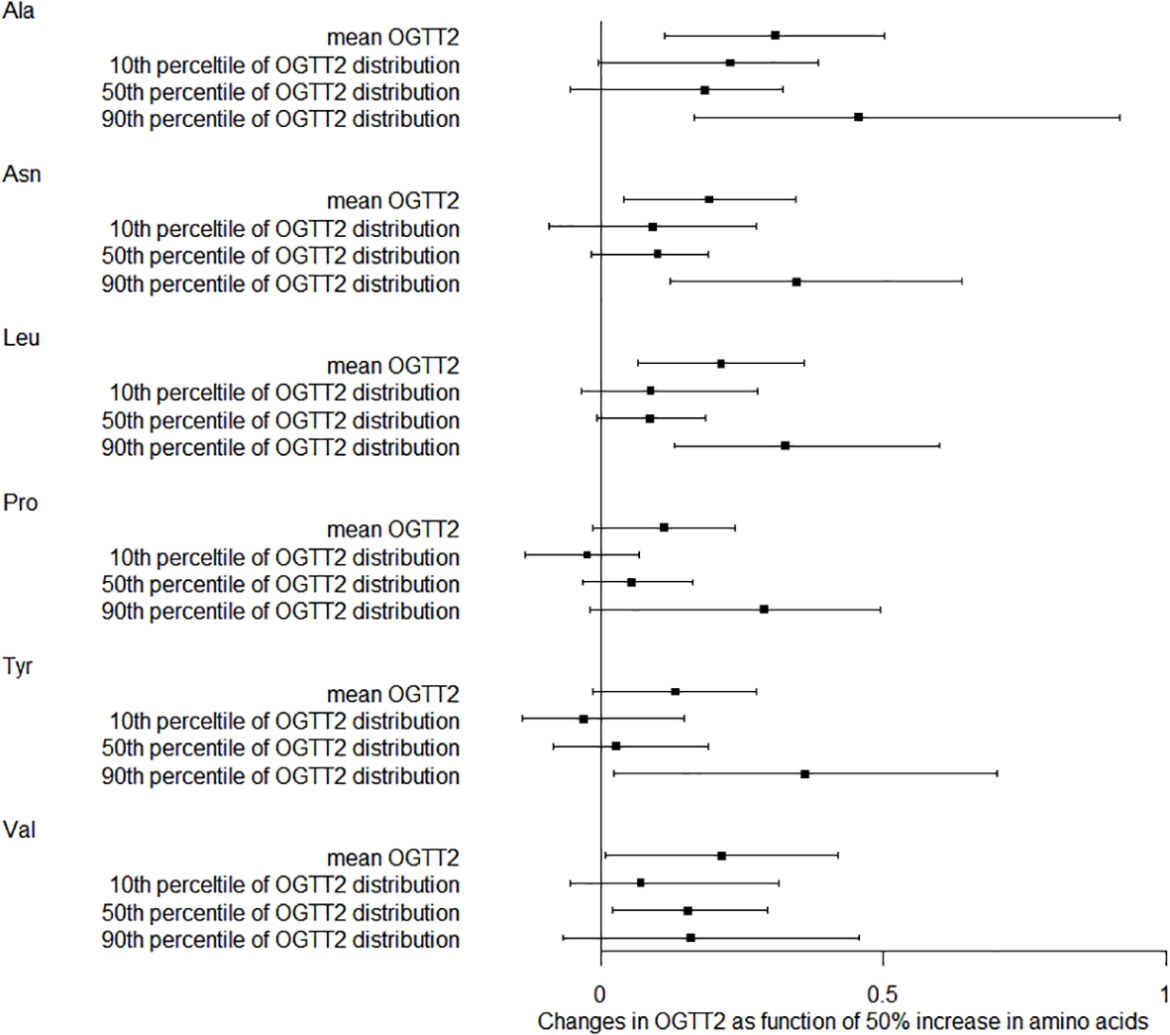
Distribution changes in OGTT2 as function of 50% increase in amino acids. The models were adjusted for maternal age, parity, level of education, family income, family history of diabetes, polycystic ovary syndrome, passive smoking, folic acid supplements prepare for pregnancy, pre-pregnancy BMI, fasting blood for amino acid metabolism, weight gain during pregnancy before OGTT, gestational age for amino acids metabolism detection.

We also studied how amino acids relate to the distribution shifts of glucose in OGTT. The 10th, 50th, and 90th percentiles for OGTT0 were 4.01, 4.39, and 4.91 mmol/L, respectively. For OGTT1, these percentiles were 5.63, 7.77, and 10.19 mmol/L, and for OGTT2, they were 5.21, 6.60, and 8.78 mmol/L. In **Figure 1**, a 50% increase in Ala levels was linked to a 0.07 mmol/L (95% CI: 0.02-0.13) rise in OGTT0 after adjustment. Importantly, this association remained statistically significant at the 90th percentile of the OGTT0 distribution, with an increase of 0.15 mmol/L (95% CI: 0.06-0.24). In contrast, no significant associations were identified between other essential amino acids and OGTT0. **Figure 2** demonstrates a significant correlation between a 50% increase in Asn and Leu levels and an increase in mean OGTT1 of 0.24 mmol/L (95% CI: 0.05-0.43) and 0.24 mmol/L (95% CI: 0.06-0.43), respectively. Additionally, a significant association was observed with Leu at the lower tail of the 10th percentile (0.15 mmol/L (95% CI: 0.01-0.32)). **Figure 3** revealed that a 50% elevation in Ala, Asn, and Leu levels corresponded to a rise of 0.31 mmol/L (95% CI: 0.11-0.50), 0.19 mmol/L (95% CI: 0.04-0.34), and 0.21 mmol/L (95% CI: 0.06-0.36) in mean OGTT2, respectively. These associations were statistically significant at the upper 90th percentile of the OGTT2 distribution, with increases of 0.46 mmol/L (95% CI: 0.16-0.92) for Ala, 0.35 mmol/L (95% CI: 0.12-0.64) for Asn, and 0.33 mmol/L (95% CI: 0.13-0.60) for Leu. Furthermore, a 50% increase in Val level was linked to a mean OGTT2 increase of 0.22 mmol/L (95% CI: 0.01-0.42).

### 4.4 Mediating effects of amino acids on BMI and glucose

**Figure 4:**
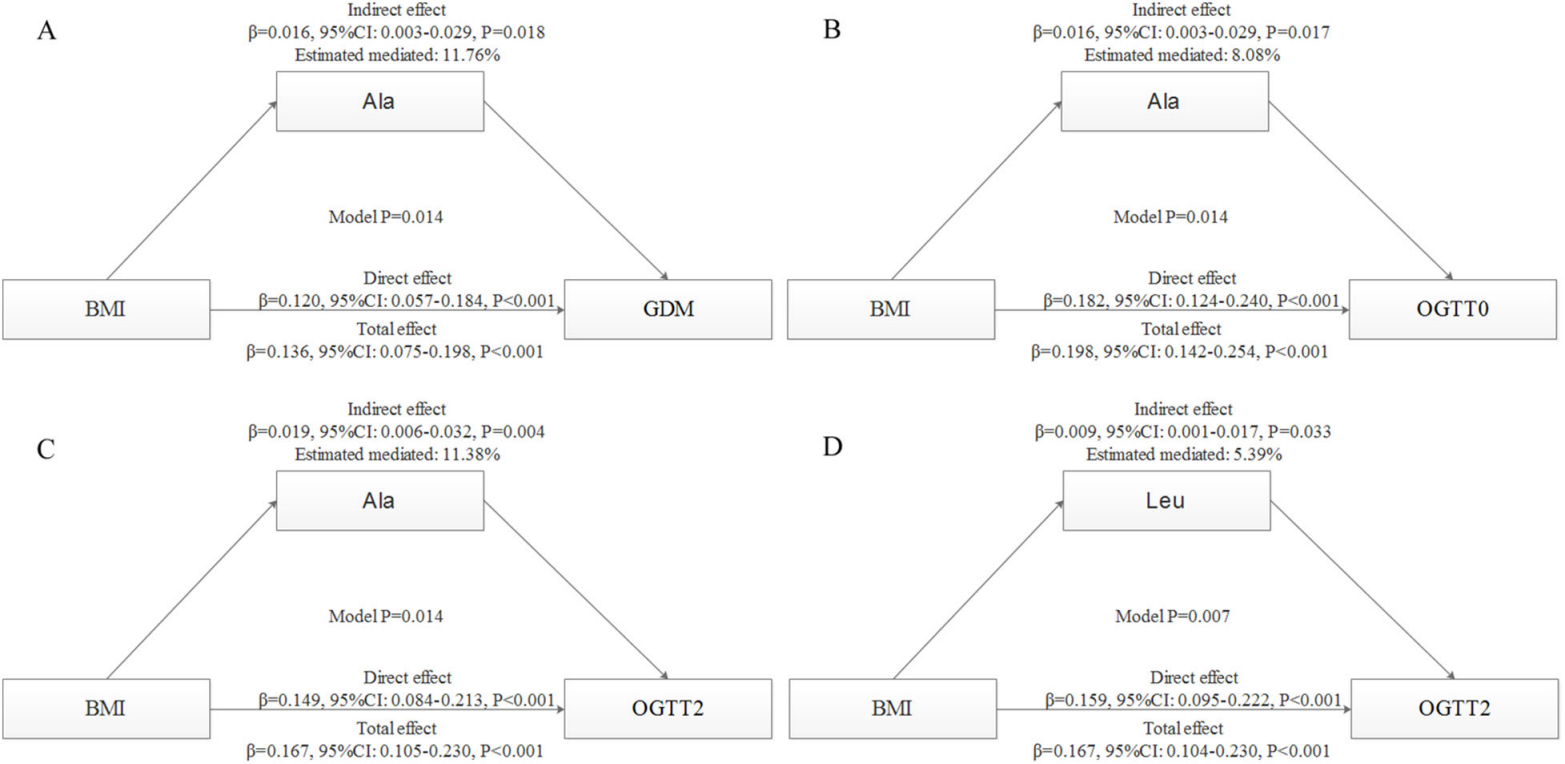
Mediation analysis with standardized coefficients of BMI, amino acids and GDM or glucose. The models were adjusted for maternal age, parity, level of education, family income, family history of diabetes, polycystic ovary syndrome, passive smoking, folic acid supplements prepare for pregnancy, fasting blood for amino acid metabolism, weight gain during pregnancy before OGTT, gestational age for amino acids metabolism detection.All models were found to be statistically significant (P < 0.05). **(A)**, mediating effects of Ala on BMI and GDM; **(B)**, mediating effects of Ala on BMI and OGTT0; **(C)**, mediating effects of Ala on BMI and OGTT2; **(D)**, mediating effects of Leu on BMI and OGTT2.

Given the observed significant correlations between BMI and GDM/glucose, a mediation analysis was conducted to investigate the potential mediating effects of amino acids on these relationships (**Figure 4**). After adjustment, the findings indicated that the Ala level accounted for 11.76% (indirect effect: 0.016, 95%CI: 0.003-0.029, P = 0.018), 8.08% (indirect effect: 0.016, 95%CI: 0.003-0.029, P = 0.017), and 11.38% (indirect effect: 0.019, 95%CI: 0.006-0.032, P = 0.004) of the associations between BMI and GDM, OGTT0, and OGTT2, respectively. A significant indirect effect of BMI associated with OGTT2 (0.009, 95%CI: 0.001-0.017, P = 0.033) via Leu level was also identified, with the estimated proportion of mediating effect being 5.39%. This implies that higher maternal BMI is associated with increased GDM incidence and glucose level, and BMI can further elevate GDM incidence and glucose level through higher amino acid concentration.

We conducted additional analyses to examine interactions between BMI and amino acids (specifically Ala and Leu); however, no significant multiplicative effects were detected, with P-values of 0.834 for BMI-Ala and 0.377 for BMI-Leu.

## 5 Discussion

This study shows a strong link between amino acid metabolism and BMI, GDM, and glucose levels. Specifically, the levels of alanine and leucine emerge as crucial mediators in the relationship between BMI and GDM or glucose intolerance. These results provide a new direction for understanding why overweight or obese mothers are more likely to have GDM and glucose intolerance.

The correlation between human metabolism and obesity and overweight is well-established. However, the relationship between BMI and amino acids remains uncertain. A recent study found that obese individuals exhibited elevated levels of leucine, isoleucine, valine, tyrosine, phenylalanine, glutamate, lysine, and alanine, while glycine levels were decreased (22). Additionally, a Chinese study found a positive association between phenylalanine, alanine, glutamic acid, tyrosine, leucine, valine, and children’s obesity, while glutamine and histidine were negatively associated (19). Our investigation results partially disagree with their research findings, possibly due to differences in our study populations. Specifically, our study focused on pregnant women, where pregnancy may affect hormone levels and alter the relationship between BMI and amino acid levels. Amino acids exert various metabolic functions within the human body, including the synthesis of tissue proteins, acids, hormones, antibodies, creatine, and other ammonia-containing substances. Additionally, they can be converted into carbohydrates and fat, oxidized to produce energy in the form of carbon dioxide, water, and urea. Consequently, a discernible relationship exists between BMI and metabolic function, necessitating further exploration of the underlying mechanisms.

GDM is a metabolic disorder that necessitates the identification of metabolic markers to effectively manage blood glucose levels in early pregnancy. Amino acids, crucial for sustaining human physiological functions, also play a significant role in insulin secretion (IR) signal transduction and glucose metabolism (23). One study demonstrated notable variations in six amino acids (serine, proline, leucine/isoleucine, glutamic acid, tyrosine, and ornithine) in individuals with GDM compared to healthy controls, suggesting a potential role of these metabolites in the development and progression of GDM (24). Another study observed a significant decrease in glycine levels and an increase in valine, leucine, phenylalanine, and combined glutamine and glutamate levels in subjects with insulin resistance (25). BCAAs were found to be elevated in patients with GDM, while AAAs did not exhibit a significant alteration (26). Contrary to their findings, our study revealed an association between GDM and leucine, valine, alanine, aspartic acid, proline, and tyrosine, which was not entirely congruent with their results. Research has indicated that BCAAs are primarily metabolized in muscle tissue to generate energy, while arginine is closely linked to both the urea cycle and immune function (27). Tyrosine is synthesized from the essential amino acid phenylalanine, and heightened tyrosine concentrations may be attributed to increased activity of tyrosine transaminase resulting from elevated insulin secretion (28). Leucine, glutamine, and alanine contribute to the hypersensitivity of amino acids in islets (29). Valine, a branched-chain amino acid, is known to be associated with insulin resistance (30). In summary, alterations in plasma amino acid concentrations are bound to induce modifications in the corresponding biochemical metabolism and physiological processes of the organism; however, the precise mechanism remains elusive. The intricate perturbation of amino acids during the progression of GDM necessitates additional research.

The observed imbalance in participant distribution across BMI categories is consistent with the natural weight distribution among Chinese women of reproductive age (31). Although the smaller sample size within the high BMI group may constrain the statistical power for subgroup analyses, our regression models have been adjusted for covariates to mitigate potential bias. Importantly, the absence of significant differences in amino acid levels between individuals with GDM and those without in the high BMI group may be attributed to two factors: (1) limited statistical power resulting from the small GDM subgroup in this category, and (2) metabolic heterogeneity in obesity, where compensatory mechanisms, such as adipose tissue buffering of amino acids, could diminish their association with glucose dysregulation (32, 33). These findings highlight the necessity for larger cohorts to validate the mediating role of amino acids in populations with high BMI and to investigate obesity-specific metabolic adaptations that may influence these relationships.

IR serves as the underlying mechanism for metabolic disorders and complications associated with obesity. A study demonstrated that obese women with GDM exhibited elevated levels of BCAAs including valine, leucine, and isoleucine in early pregnancy, in comparison to non-obese women with GDM (16). Our findings also indicated a significant positive correlation between BMI and concentrations of alanine and leucine, which aligns partially with previous research. Furthermore, we have identified alanine and leucine as potential mediators in the relationship between BMI and blood glucose levels. This discovery offers valuable insights for future investigations into the interplay among these three variables. It has been observed that obesity and IR have a diminishing effect on the expression levels of branched-chain amino acid aminotransferase and branched-chain amino acid keto acid dehydrogenase, consequently impeding the breakdown of BCAAs. Conversely, IR enhances protein breakdown, leading to an increased production of BCAAs. The elevated levels of BCAAs further exacerbate IR, thereby exacerbating the progression and manifestation of the aforementioned process (34). Hence, it is plausible that overweight and obesity exert a multifaceted impact on amino acid metabolism, potentially resulting in the development of IR. Consequently, additional research is warranted to explore the correlation between BMI, alanine/leucine levels, and blood glucose fluctuations throughout pregnancy.

However, we recognize that dietary and environmental factors can influence amino acid levels. Although a standardized diet was not implemented in this study, we accounted for the covariate of fasting status during blood sample collection to mitigate potential bias in our analysis. Furthermore, our hospital does not mandate fasting for pregnant women at their initial prenatal examination, reflecting real-world practices. Consequently, the lack of fasting requirements enhances the applicability and generalizability of our findings. Additionally, we controlled for various environmental factors, including education level, household income, exposure to secondhand smoke, and folic acid intake, which significantly mitigated potential confounding effects. Nonetheless, it is important to acknowledge the absence of dietary survey data in this study, such as long-term dietary patterns (e.g., high intake of branched-chain amino acids), which may influence baseline amino acid levels. Furthermore, the impact of environmental factors, such as exposure to pollutants, on metabolism warrants further investigation. In our forthcoming study, we will incorporate a dietary assessment module to examine the relationship between the daily dietary habits of pregnant women, amino acid metabolism, and GDM.

This study possesses several notable strengths. Despite numerous studies indicating a correlation between amino acids and the risk of GDM or IR, scholars remain divided on the causal connection between amino acids and GDM. The question of whether amino acids serve as the catalyst for GDM or merely function as biomarkers for metabolic disorders associated with GDM remains unresolved. However, this study stands out as a prospective birth cohort study with a substantial sample size, thereby enhancing the accuracy and reliability of the evidence pertaining to relevant causality. Furthermore, the utilization of mediation model analysis revealed that alanine and leucine play pivotal roles as mediators between BMI and glucose or GDM. This investigation aims to identify characteristic amino acids associated with GDM for early clinical prediction and the identification of high-risk populations, thereby offering valuable insights into the underlying etiological mechanism and serving as a foundation for future targeted intervention studies. Finally, by incorporating a wide range of BMI samples, this study systematically elucidates the continuous association between pre-pregnancy BMI and amino acid levels, thereby providing a more universal metabolic target for early intervention in GDM.

Nevertheless, it is important to acknowledge the limitations of our study. Firstly, the diagnosis of GDM was predominantly made during the second trimester, potentially overlooking the possibility that hyperglycemia could have commenced prior to pregnancy, thereby influencing the amino acid profile to already reflect IR. Secondly, the measurement of amino acids at a singular timepoint may not adequately capture the dynamic fluctuations that transpire throughout the course of pregnancy. Consequently, additional research is imperative to ascertain the most suitable timeframe for measuring amino acids. In addition, the presence of circulating amino acids may serve as an indicator of the sustained intake of dietary amino acids, particularly when considering the plasma levels of essential amino acids, which exhibit a strong correlation with one’s habitual dietary patterns. Moving forward, it is imperative to undertake comprehensive investigations on dietary practices in order to delve deeper into the intricate connection between amino acids and blood glucose levels. Finally, the inclusion of women with PCOS may introduce metabolic variability; however, after statistical adjustments, the results consistently revealed independent associations between BMI and amino acid levels. Future research should consider further stratifying analyses to distinguish between PCOS and non-PCOS populations.

In summary, the metabolic characteristics of amino acids exhibit a noteworthy correlation with BMI, GDM, and glucose levels during the pregnancy. Particularly, the concentrations of alanine and leucine play a substantial role in mediating the association between BMI and GDM or glucose intolerance. Further research is necessary to confirm the potential mechanisms involved. Additionally, heightened attention should be given to individuals with overweight and obesity and elevated plasma amino acid levels, as they are more prone to developing GDM. Simultaneously, additional intervention studies are required to investigate whether reducing alanine and leucine concentrations can affect outcomes in overweight and obese pregnant women who are at a heightened risk of GDM.

## Data Availability

The raw data supporting the conclusions of this article will be made available by the authors, without undue reservation.
