# Supplementary material for: Exploring the mediating effects of amino acids on BMI and gestational diabetes mellitus: a longitudinal population-based cohort study
Source: Front Endocrinol (Lausanne). 2025 May 20;16:1502678. doi: 10.3389/fendo.2025.1502678 (PMC12129754; doi:10.3389/fendo.2025.1502678)
Supplement: Supplementary file 1 [file Table1.docx]

**Table A1** Differences of amino acids between GDM and non-GDM in BMI subgroups

| Amino acids  (mean ± SD) | BMI<18.5  (n = 256) | | | 18.5 ≤ BMI<24  (n = 693) | | | BMI ≥ 24  (n = 125) | | |
| --- | --- | --- | --- | --- | --- | --- | --- | --- | --- |
|  | non-GDM | GDM | P-value | non-GDM | GDM | P-value | non-GDM | GDM | P-value |
| Essential amino acid (μmol/L) |  |  |  |  |  |  |  |  |  |
| His | 125.42 ± 60.96 | 108.74 ± 35.92 | 0.138 | 130.46 ± 78.96 | 125.31 ± 61.45 | 0.357 | 123.71 ± 46.15 | 128.89 ± 56.62 | 0.584 |
| Leu | 92.01 ± 24.53 | 91.65 ± 18.82 | 0.938 | 93.44 ± 22.48 | 99.58 ± 24.26 | **0.004*** | 100.24 ± 27.04 | 102.93 ± 22.74 | 0.581 |
| Lys | 25.08 ± 23.77 | 23.40± 6.98 | 0.697 | 25.81 ± 25.22 | 25.23 ± 25.71 | 0.570 | 24.65 ± 13.95 | 33.17 ± 37.49 | 0.068 |
| Met | 16.49 ± 4.66 | 15.49± 4.18 | 0.261 | 16.24 ± 4.13 | 16.47 ± 4.87 | 0.394 | 15.72 ± 3.23 | 17.52 ± 4.06 | **0.008*** |
| Thr | 14.57 ± 3.32 | 14.79 ± 3.01 | 0.293 | 15.49 ± 3.39 | 15.73 ± 3.50 | 0.293 | 16.56 ± 3.07 | 15.95 ± 4.20 | 0.357 |
| Trp | 52.93 ± 11.76 | 48.76 ± 8.21 | 0.405 | 51.88 ± 13.25 | 52.21 ± 10.86 | 0.405 | 50.96 ± 9.15 | 50.25 ± 11.15 | 0.704 |
| Val | 147.16 ± 27.60 | 142.33 ± 25.55 | 0.358 | 150.70 ± 26.48 | 159.56 ± 30.04 | **0.001*** | 158.66 ± 27.36 | 162.17 ± 27.62 | 0.501 |
| Non-essential amino acid (μmol/L) |  |  |  |  |  |  |  |  |  |
| Ala | 138.94 ± 25.49 | 141.44 ± 25.81 | 0.609 | 146.30 ± 27.47 | 153.91 ± 28.33 | **0.005** | 153.07 ± 27.41 | 161.80 ± 27.62 | 0.096 |
| Arg | 16.49 ± 8.76 | 13.18 ± 6.21 | **0.043*** | 15.41 ± 6.97 | 15.37 ± 7.31 | 0.948 | 17.31 ± 7.01 | 16.98 ± 7.95 | 0.812 |
| Asn | 14.69 ± 3.56 | 14.60 ± 2.92 | 0.898 | 15.12 ± 3.62 | 16.07 ± 3.81 | **0.008*** | 16.34 ± 4.29 | 16.70 ± 3.21 | 0.634 |
| Asp | 37.16 ± 11.87 | 33.36 ± 9.21 | 0.088 | 36.65 ± 10.86 | 36.42 ± 10.93 | 0.834 | 41.32 ± 14.11 | 35.39 ± 13.40 | **0.028*** |
| Cys | 0.46 ± 0.17 | 0.49 ± 0.15 | 0.438 | 0.47 ± 0.21 | 0.50 ± 0.15 | 0.128 | 0.46 ± 0.14 | 0.43 ± 0.15 | 0.352 |
| Gln | 27.87 ± 27.35 | 25.38 ± 6.78 | 0.615 | 28.72 ± 28.97 | 28.02 ± 29.67 | 0.807 | 26.93 ± 13.18 | 37.40 ± 43.48 | 0.134 |
| Glu | 78.87 ± 14.60 | 77.78 ± 14.24 | 0.696 | 79.23 ± 14.25 | 80.44 ± 15.59 | 0.393 | 81.45 ± 14.09 | 79.79 ± 15.53 | 0.550 |
| Gly | 84.95 ± 18.05 | 87.18 ± 21.66 | 0.529 | 86.19 ± 18.39 | 86.69 ± 22.51 | 0.789 | 86.61 ± 20.67 | 82.92 ± 17.50 | 0.323 |
| Phe | 44.06 ± 8.73 | 43.00 ± 7.12 | 0.520 | 45.66 ± 8.61 | 46.41 ± 8.61 | 0.381 | 47.23 ± 12.65 | 49.13 ± 9.84 | 0.438 |
| Pro | 419.73 ± 121.64 | 431.75 ± 119.26 | 0.606 | 442.47 ± 126.25 | 480.86 ± 141.67 | **0.002*** | 457.73 ± 128.01 | 493.80 ± 152.16 | 0.166 |
| Ser | 71.72 ± 16.50 | 71.81 ± 15.87 | 0.977 | 71.78 ± 17.34 | 71.75 ± 16.69 | 0.987 | 75.92 ± 20.26 | 68.15 ± 22.18 | 0.052 |
| Tyr | 29.78 ± 8.43 | 28.78 ± 5.70 | 0.730 | 29.85 ± 7.57 | 31.45 ± 7.88 | **0.032*** | 32.94 ± 8.91 | 33.56 ± 6.64 | 0.357 |
| Metabolism of amino acid (μmol/L) |  |  |  |  |  |  |  |  |  |
| Cit | 14.71 ± 4.31 | 14.29 ± 2.97 | 0.600 | 14.32 ± 3.39 | 14.78 ± 3.28 | 0.162 | 14.18 ± 3.10 | 14.92 ± 3.67 | 0.237 |
| Hcy | 13.89 ± 1.49 | 14.19 ± 1.21 | 0.290 | 13.76 ± 1.45 | 13.71 ± 1.46 | 0.664 | 13.83 ± 1.48 | 13.33 ± 1.72 | 0.093 |
| Orn | 19.13 ± 4.27 | 17.65± 4.09 | 0.071 | 19.47 ± 4.39 | 19.25 ± 4.19 | 0.605 | 20.73 ± 4.90 | 19.28 ± 4.46 | 0.111 |
| Pip | 95.47 ± 18.72 | 96.79 ± 26.60 | 0.729 | 95.49 ± 20.12 | 95.39 ± 20.88 | 0.958 | 95.68 ± 19.37 | 92.94 ± 17.12 | 0.438 |

T-test was utilized to compare women with and without GDM. *P<0.05. GDM, gestational diabetes mellitus; SD, standard deviation; BMI, body mass index; OGTT, oral glucose tolerance test.

**Table A2** Association between amino acids and the risk of GDM based on three different amino acids levels.

| Amino acids | Crude Model | P-value | Adjusted Model^a^ | P-value |
| --- | --- | --- | --- | --- |
|  | OR (95% CI) |  | OR (95% CI) |  |
| Leu (μmol/L) |  |  |  |  |
| 1st tertile | 1 (ref) | - | 1 (ref) | - |
| 2nd tertile | 1.20 (0.81-1.79) | 0.363 | 1.25 (0.83-1.90) | 0.280 |
| 3rd tertile | 1.75 (1.20-2.56) | **0.004*** | 1.53 (1.02-2.28) | **0.038*** |
| Val (μmol/L) |  |  |  |  |
| 1st tertile | 1 (ref) | - | 1 (ref) | - |
| 2nd tertile | 1.56 (1.05-2.32) | **0.028*** | 1.49 (0.98-2.26) | 0.062 |
| 3rd tertile | 1.87 (1.27-2.77) | **0.002*** | 1.60 (1.05-2.43) | **0.028*** |
| Ala (μmol/L) |  |  |  |  |
| 1st tertile | 1 (ref) | - | 1 (ref) | - |
| 2nd tertile | 1.31 (0.88-1.96) | 0.187 | 1.26 (0.83-1.92) | 0.285 |
| 3rd tertile | 1.93 (1.32-2.83) | **0.001*** | 1.62 (1.08-2.43) | **0.020*** |
| Asn (μmol/L) |  |  |  |  |
| 1st tertile | 1 (ref) | - | 1 (ref) | - |
| 2nd tertile | 1.75 (1.17-2.63) | **0.007*** | 1.74 (1.14-2.66) | **0.010*** |
| 3rd tertile | 2.17 (1.46-3.22) | **<0.001*** | 1.91 (1.26-2.90) | **0.002*** |
| Pro (μmol/L) |  |  |  |  |
| 1st tertile | 1 (ref) | - | 1 (ref) | - |
| 2nd tertile | 1.32 (0.88-1.94) | 0.190 | 1.23 (0.81-1.87) | 0.327 |
| 3rd tertile | 1.83 (1.25-2.68) | **0.002*** | 1.69 (1.12-2.55) | **0.012*** |
| Tyr (μmol/L) |  |  |  |  |
| 1st tertile | 1 (ref) | - | 1 (ref) | - |
| 2nd tertile | 1.61 (1.08-2.38) | **0.018*** | 1.61 (1.08-2.40) | **0.020*** |
| 3rd tertile | 1.72 (1.16-2.54) | **0.007*** | 1.62 (1.09-2.43) | **0.018*** |

Logistic regression was utilized to explore the association. *P<0.05. GDM, gestational diabetes mellitus.

^a^ adjusted for maternal age, parity, level of education, family income, family history of diabetes, polycystic ovary syndrome, passive smoking, folic acid supplements prepare for pregnancy, pre-pregnancy BMI, fasting blood for amino acid metabolism, weight gain during pregnancy before OGTT, and gestational age for amino acids metabolism detection.
